# Mechanism patterns and age variations in pediatric cranio-maxillofacial trauma: a 5-year analysis of the national trauma data bank

**DOI:** 10.1007/s10006-025-01450-3

**Published:** 2025-09-16

**Authors:** Kirav Patel, Bryce Thornton, Boyu Ma, Yedeh Ying, Jaime Castro-Nunez

**Affiliations:** 1 Department of Oral and Maxillofacial Surgery, University of Alabama at Birmingham, Birmingham, AL USA; 2 School of Dentistry, University of Alabama at Birmingham, Birmingham, AL USA

**Keywords:** Facial injuries, Facial bones, Fracture fixation, Pediatrics, Child, Databases, Factual, Infant, Mandibular fractures, Maxillary fractures, Zygomatic fractures, Orbital fractures, Nasal bone, Skull fracture, Skull fracture, Basilar, Hematoma, Epidural, Cranial, Cranial nerve injuries, Mandibular condyle

## Abstract

**Purpose:**

Pediatric craniomaxillofacial trauma presents unique diagnostic and management challenges due to the anatomical and developmental characteristics of children.

**Methods:**

This retrospective study analyzed 795,431 pediatric trauma cases (ages 0–18 years) from the National Trauma Data Bank (2017–2022) to characterize injury patterns and risk factors.

**Results:**

Among 119,324 cases (15.0%) involving craniomaxillofacial fractures, incidence increased significantly with age, from 0.93% in infants to 5.59% in adolescents. Motor vehicle collisions were the leading mechanism, doubling fracture risk (odds ratio 2.39, 95% confidence interval 2.19–2.59), while proper restraint use reduced risk by 43% (odds ratio 0.572, 95% confidence interval 0.52–0.62). Falls were the predominant mechanism in younger children, whereas motor vehicle collisions, assault, and firearm-related injuries increased with age. Fracture patterns shifted developmentally: cranial vault fractures dominated in younger children, while cranial base, midface, mandibular, and dental fractures became prevalent in older populations.

**Conclusion:**

These findings emphasize the need for age-specific prevention strategies, including improved restraint compliance and targeted injury mitigation programs, to reduce the burden of pediatric facial trauma.

**Supplementary Information:**

The online version contains supplementary material available at 10.1007/s10006-025-01450-3.

## Introduction

Craniomaxillofacial (CMF) trauma in children encompasses a diverse range of injuries with unique challenges that arise from the anatomical and developmental characteristics of the pediatric population [1–3]. Compared to adults, children have an underdeveloped facial skeleton, a proportionately larger cranial size, incompletely developed paranasal sinuses, which provides some protection against fractures but also influences injury patterns sustained [3, 4]. While pediatric CMF injuries are less frequent compared to those in adults, they often carry significant functional, aesthetic, and psychosocial implications [5, 6]. The potential for associated injuries, particularly intracranial trauma, further underscores the complexity of diagnosing and managing these cases effectively [7].

Previous studies on pediatric CMF trauma rely on limited, single-institution data, leading to inconsistencies in reported patterns of injury and management approaches [5, 8, 9]. Furthermore, these studies lack the ability to examine population-level trends or to account for variations based on developmental stages or regional differences [9, 10]. Advances in public health interventions and technology, such as improvements in motor vehicle safety, have also influenced trauma mechanisms, necessitating updated analyses of how these changes impact pediatric injury patterns [11, 12]. Despite these improvements, large-scale, multicenter analyses remain scarce, leaving critical gaps in understanding the factors driving injury patterns, their outcomes, and contemporary management strategies [9, 13–15].

The purpose of this study is to investigate the epidemiology of pediatric CMF trauma using data from the National Trauma Data Bank (NTDB), one of the largest repositories of trauma cases in the United States. We hypothesize there are specific patterns to fractures according to the demographics and mechanism. Specifically, our aims were to (1) characterize the demographic and clinical profiles of pediatric CMF trauma patients, (2) examine the mechanisms of injury and their distribution across age groups, and (3) evaluate the prevalence and patterns of associated injuries. By addressing these objectives, the study seeks to identify developmental, demographic, and mechanism-specific variations in injury patterns. Ultimately, these findings aim to improve patient outcomes by advancing the understanding of pediatric CMF trauma and evidence-based care strategies for this vulnerable population.

## Methods

### Study design and sample

This retrospective cohort study analyzed pediatric trauma cases from the National Trauma Data Bank (NTDB) between 2017 and 2022. The NTDB, maintained by the American College of Surgeons Committee on Trauma, is the largest trauma repository in North America containing de-identified data from trauma centers across the United States [14]. The database includes covering patient demographics, injury characteristics, procedures, and outcomes. Our inclusion criteria was pediatric trauma patients aged 0 to 18 years with CMF fractures and identified using International Classification of Diseases, Tenth Revision, Clinical Modification (ICD-10-CM) codes in accordance with the NTDB Data Dictionary. Data extraction involved standard logic checks and cleaning procedures to ensure consistency across reporting years. Our exclusion criteria were cases with missing critical demographic or clinical variables were excluded. This study was determined to be IRB exempt.

### Variables

Patients were categorized into age groups corresponding to key developmental stages: infancy (0–1 years), early childhood (2–4 years), middle childhood (5–9 years), pre-adolescence (10–14 years), and adolescence (15–18 years)—as defined by Imahara et al. [9]. Primary variables included demographic characteristics, injury mechanisms, and fracture types. CMF fracture types were classified according to specific ICD-10-CM codes.

### Statistical analysis

Descriptive statistics was used to summarize patient demographics, injury mechanisms, and diagnoses. Categorical variables were expressed as frequencies and proportions, while continuous variables were summarized using means and standard deviations for normally distributed data or medians and interquartile ranges for skewed data. Group comparisons for categorical variables were conducted using chi-square tests, and independent t-tests were applied to continuous variables. Multivariate logistic regression models were constructed to evaluate the association between motor vehicle collisions, restraint use, and the likelihood of CMF trauma. Model diagnostics, including assessments for multicollinearity and goodness-of-fit, were performed. Results are reported as odds ratios (ORs) with 95% confidence intervals (CIs), and statistical significance was defined as a two-tailed p-value < 0.05. All statistical analyses were performed using the Python libraries—pandas, SciPy, and NumPy [16–19].

## Results

During the study period, 795,431 pediatric trauma cases were recorded in the NTDB, representing 11.8% of a total of 6,715,967 trauma cases. Among these, 119,324 cases (15.0%) involved CMF fractures. Patients with CMF fractures were slightly older, predominantly males, and more frequently white (Table 1). The incidence of CMF fractures increased significantly with age, rising from 0.93% (7,391 cases) among infants (0–1 years) to 5.59% (44,501 cases) among adolescents (15–18 years) (χ² = 3444.52, df = 4, *p* < 0.001) (Supplemental Fig. 1). Injury mechanisms differed significantly between patients with and without CMF fractures (χ² = 23362.53, df = 20, *p* < 0.001). In the CMF cohort, motor vehicle collisions (MVCs) were the leading cause (34.3%), followed by falls (28.4%) and assault (14.3%) (Table 2). In contrast, falls predominated in non-fracture injuries (36.2%), while MVCs and assault accounted for 22.4% and 9.13% of cases, respectively (Table 2). Overall, MVCs represented 24.2% (192,348 cases) of pediatric trauma cases, of which, yet only 60.3% (115,935) of children were adequately restrained. Over half (56.1%) of the 5,224 pediatric MVC fatalities were unrestrained. In MVC-related trauma, 51.3% of CMF-fracture patients were unrestrained compared to 36.6% of patients without fractures. Logistic regression analysis demonstrated that MVCs more than doubled the risk of sustaining a CMF fracture (OR = 2.39, 95% CI: 2.19–2.59), while restraint use reduced this risk by 42.8% (OR = 0.572, 95% CI: 0.52–0.62). The estimated probability of sustaining a CMF fracture during an MVC was 17.5% for restrained patients versus 27.0% for unrestrained patients, reflecting an absolute risk increase of 9.50% (Table 2).

**Table 1:** Demographic and clinical characteristics of pediatric patients with and without CMF fractures

Characteristic	CMF Fractures	No CMF Fractures	*p*-Value
*n*	119,324	676,107	
Percentage of patients	15.0%	85.0%	
**Age (y)**,** mean +/- SD**	**10.8 +/- 5.7**	**10.4 +/- 5.4**	**< 0.001**
**Male (%)**	**66.8%**	**64.8%**	**< 0.001**
**White (%)**	**65.3%**	**64.0%**	**< 0.001**

**Table 2:** Distribution of injury mechanisms among patients with and without CMF fractures

Mechanism (%)	CMF Fractures	No CMF Fractures	*p*-Value
MVC	34.3%	22.4%	< 0.001
Fall	28.4%	36.2%	< 0.001
**Assault**	**14.3%**	**9.13%**	**< 0.001**
**Firearm**	**5.04%**	**5.87%**	**< 0.001**
**Bicycle**	**4.63%**	**3.33%**	**< 0.001**
**Pedestrian**	**1.44%**	**0.97%**	**< 0.001**
**Bites**	**1.28%**	**3.45%**	**< 0.001**
**Cut/Pierce**	**0.40%**	**3.87%**	**< 0.001**
**Unrestrained MVC**	**51.3%**	**36.6%**	**< 0.001**

**Fig. 1:**
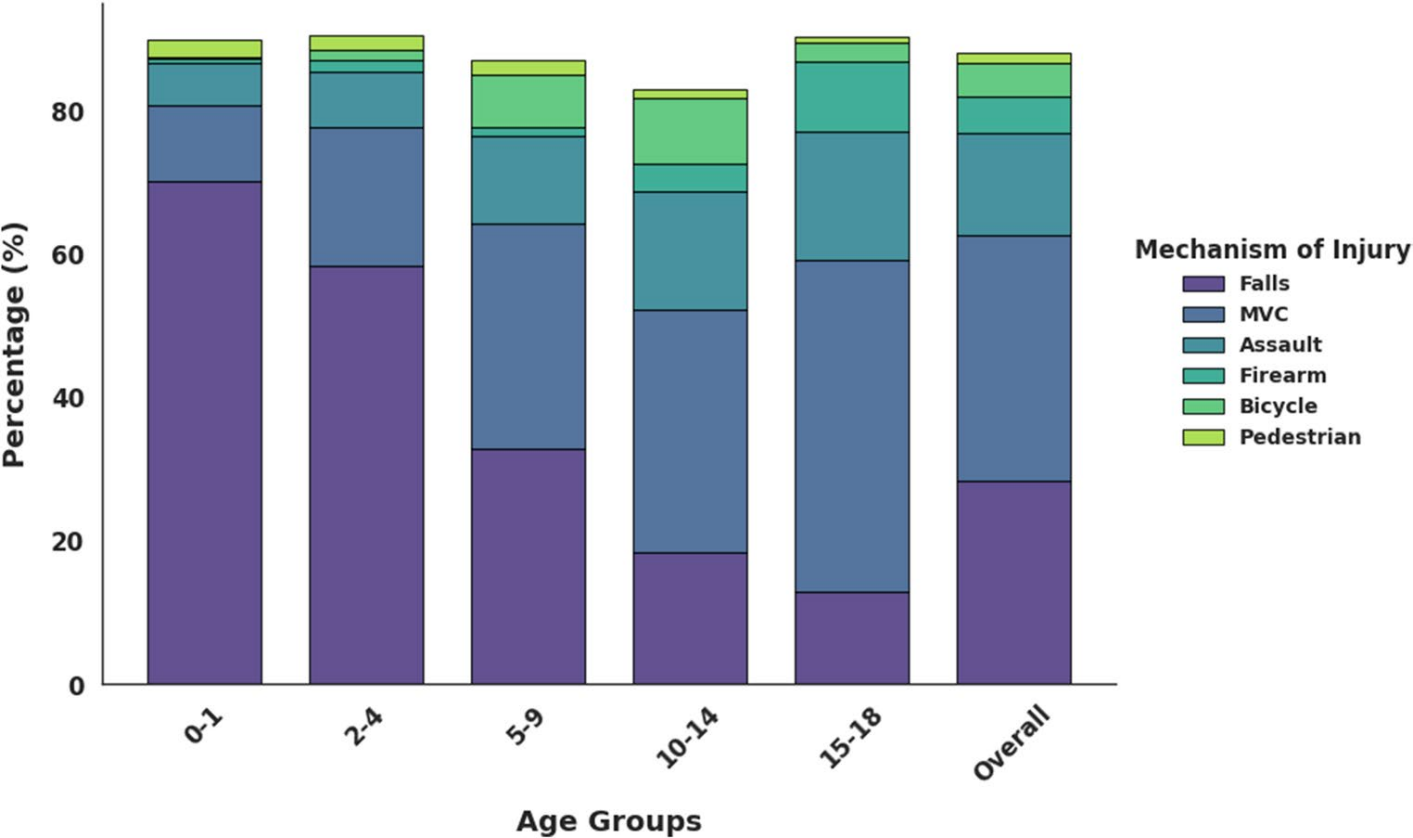
Distribution of mechanisms of CMF fractures stratified by pediatric age groups

**Fig. 2:**
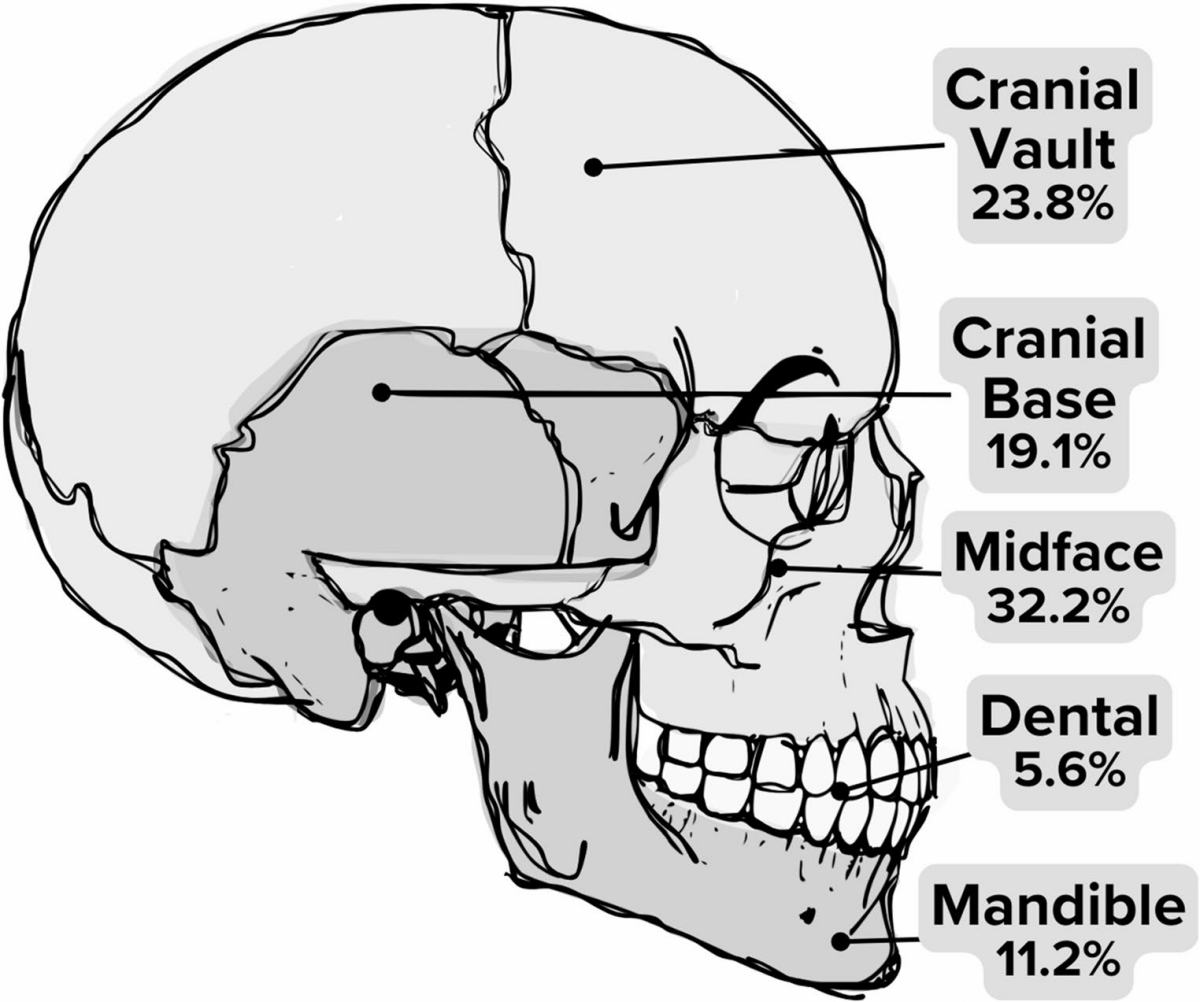
Percentage breakdown of pediatric CMF injuries by anatomic region. The midface was considered to be the maxilla, zygoma, malar process, nasal bones, and midfacial orbit (medial, lateral, and inferior walls). Not shown are other non-distinguished fractures (8.1%)

**Fig. 3:**
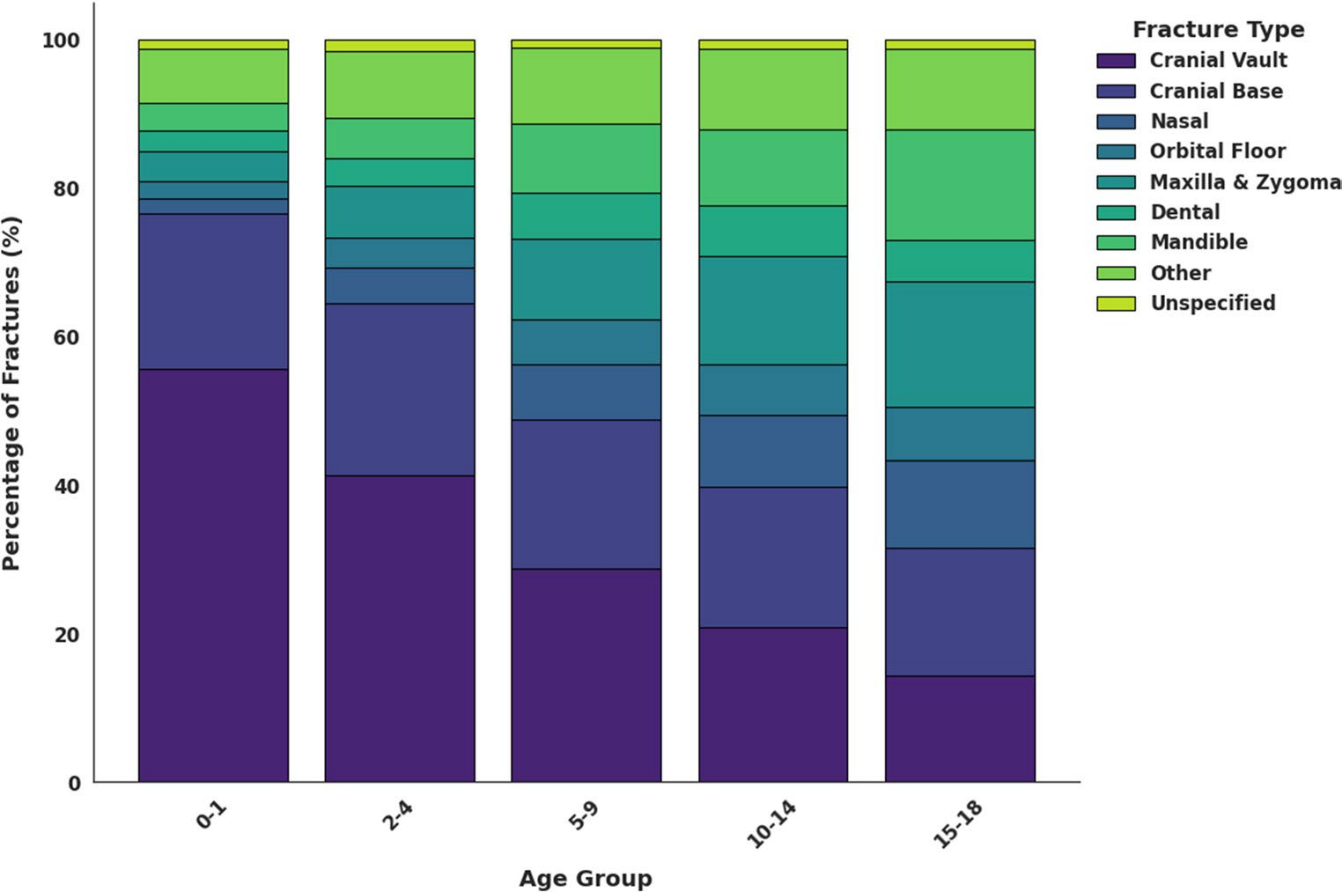
Percentage distribution of CMF fracture types across pediatric age groups. Diagnosis of fracture type made by ICD-10 classification

**Fig. 4:**
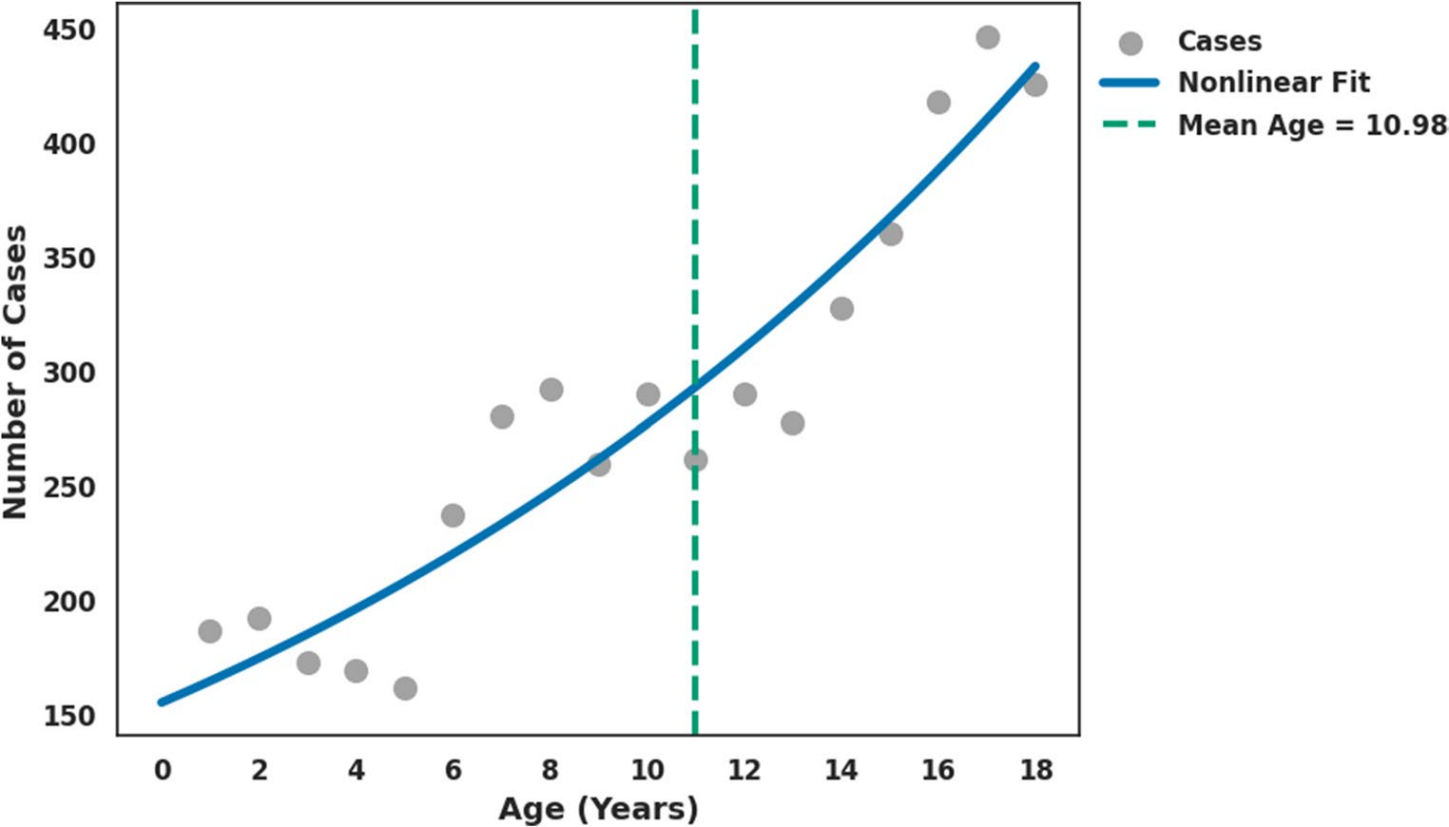
Distribution of isolated dentoalveolar fracture cases across pediatric age groups

Age-stratified analysis revealed that falls were the predominant injury in younger children, while the incidence of MVCs, assault, and firearm-related injuries increased with age. Bicycle-related injuries peaked in mid-childhood to early adolescence (Fig. 1). When fractures were grouped by facial region, midface fractures were the most common overall (Fig. 2). Analysis by ICD-10 code revealed that cranial vault fractures constituted 23.8% of all CMF fractures, followed by cranial base fractures (19.1%) and maxillary/zygoma fractures (13.4%) (Table 3). The distribution of these fracture types varied significantly across age groups (χ² = 69.20, df = 32, *p* < 0.001). Cranial vault fractures were the most frequent across all age groups, except in adolescents, who most frequently sustained cranial base fractures (Fig. 3). Fractures of the maxilla/zygoma, nasal bones, and mandible increased progressively with age (Fig. 3). Dentoalveolar fractures co-occurred with another CMF fracture in 56.4% of cases. The mean age for isolated dentoalveolar trauma was ~ 11 years, with a secondary peak in children aged 2–3 years (Fig. 4).

**Table 3:** Distribution of fracture types among patients with CMF fractures

Fracture Type	Number of Fractures	Percentage of Fractures (%)
Cranial vault	49,833	23.8
Cranial base	40,068	19.1
**Nasal bones**	**19**,**439**	**9.27**
**Orbital floor**	**12**,**952**	**6.17**
**Maxilla and zygoma**	**28**,**118**	**13.4**
**Tooth (traumatic)**	**11**,**681**	**5.57**
**Mandible**	**23**,**525**	**11.2**
**Other craniofacial bones**	**21**,**507**	**10.3**
**Unspecified craniofacial bones**	**2**,**632**	**1.25**
**Total**	**209**,**755**	

**Fig. 5:**
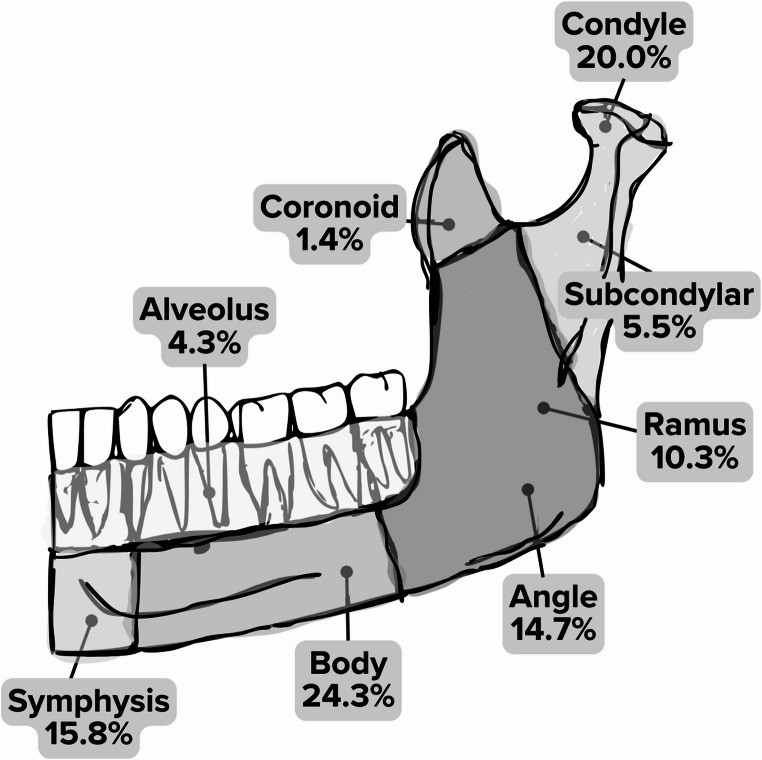
Distribution of mandibular fractures by anatomical location. Other non-distinguished fractures (3.7%) not shown

**Fig. 6:**
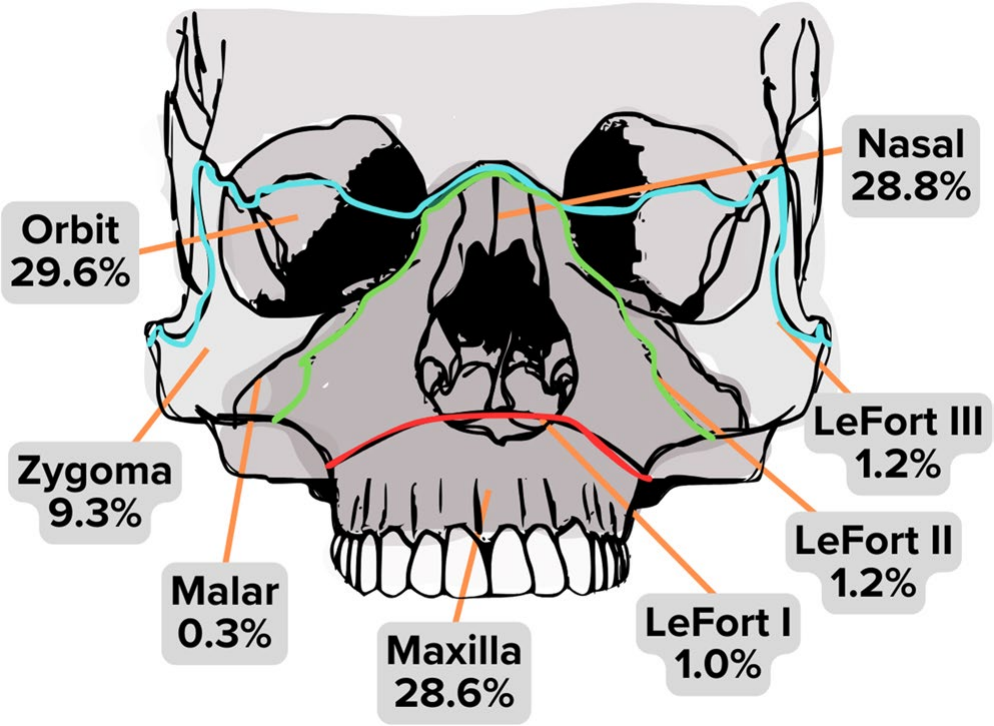
Distribution of midfacial fractures among pediatric CMF patients

**Fig. 7:**
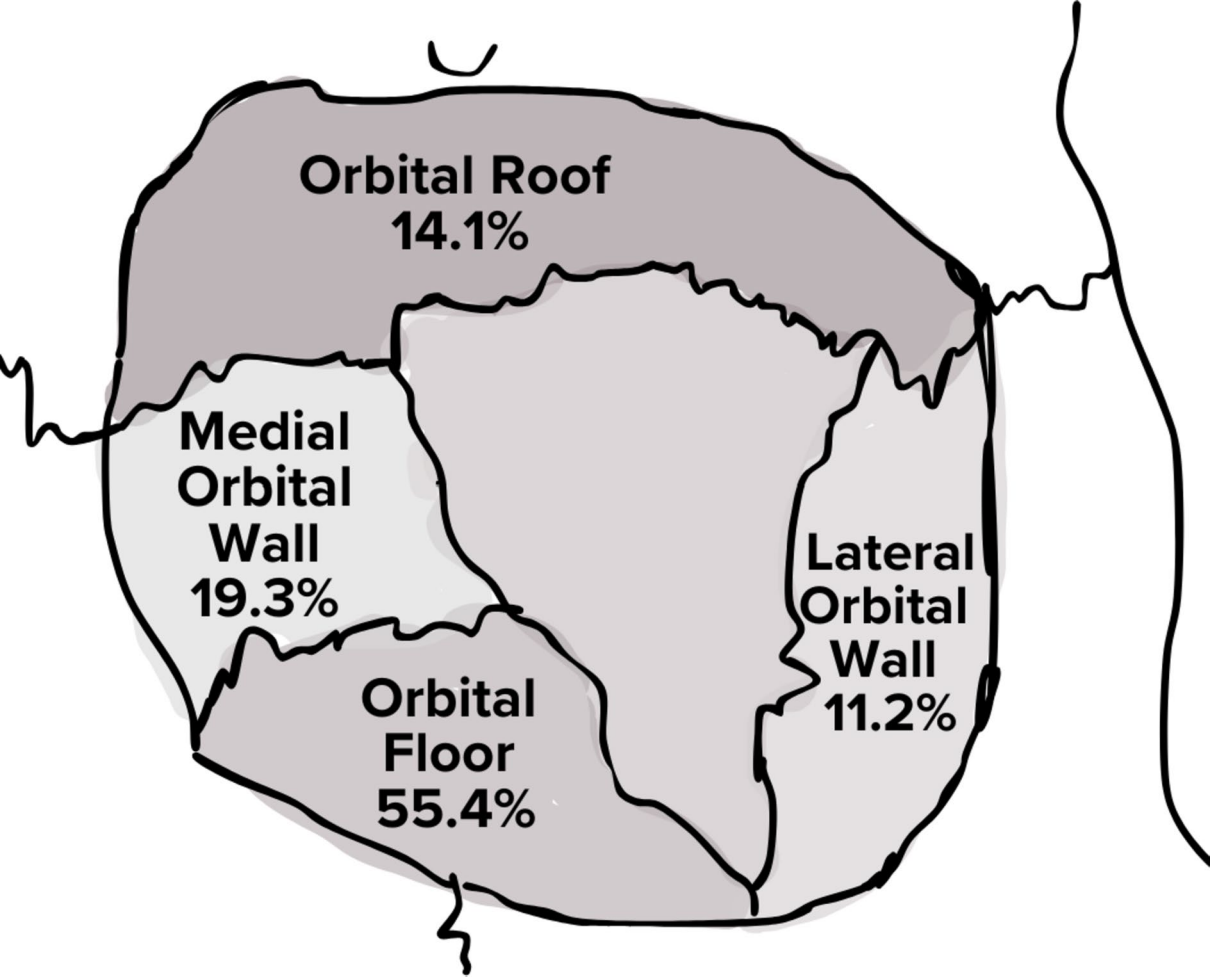
Distribution of orbital wall fractures among pediatric CMF patients

Mandibular fractures accounted for 11.2% of CMF injuries (*n* = 15,799). The body was the most frequently fractured (24.3%), followed by the condylar (20.0%) and symphyseal regions (15.8%) (Fig. 5). Multiple mandible fractures were observed in 25.9% of cases, with combined condyle and symphysis fractures being the most common pattern followed by combined symphysis and angle fractures and bilateral condylar fractures (data not shown). Among midfacial fractures (*n* = 67,499), orbital midface fractures were the most frequent (29.6%) (Fig. 6). These fractures were predominantly localized to the orbital floor (55.4%) and the medial orbital wall (19.3%), whereas injuries to the roof (14.1%) and lateral wall (11.2%) were less common (Fig. 7).

**Fig. 8:**
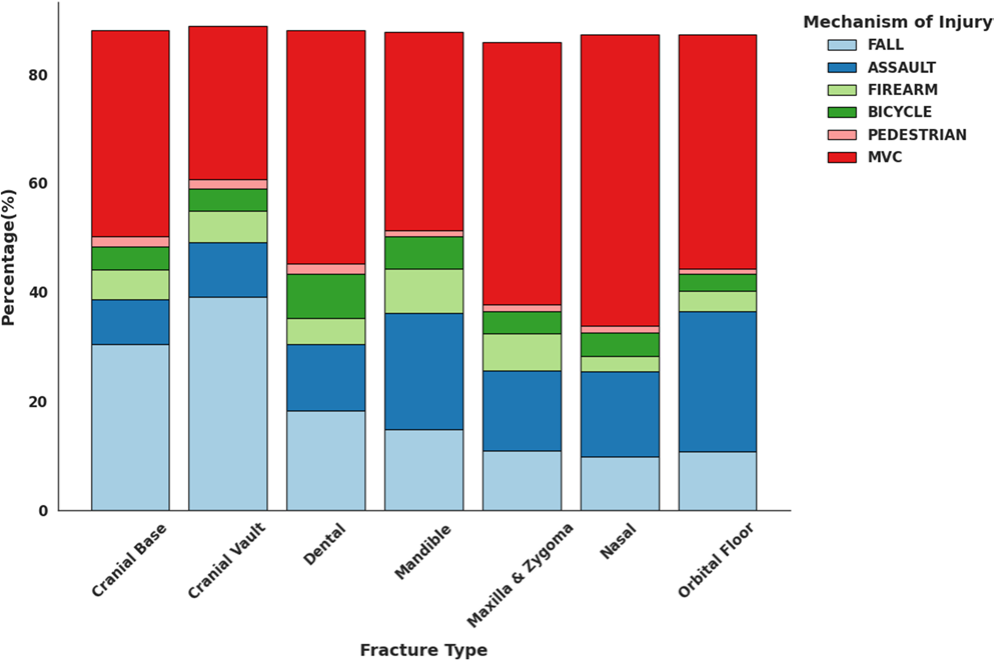
Distribution of mechanisms of injury stratified by CMF fracture type

The distribution of injury mechanisms varied significantly across CMF fracture types (χ² = 72.79, *p* < 0.001). Cranial vault fractures were most commonly associated with falls (39.1%), whereas cranial base fractures were most frequently MVC-related (37.8%) (Fig. 8). MVCs were the leading mechanism for midface fractures, with 53.6% of nasal fractures, 48.2% of maxilla/zygoma fractures, and 43.0% of orbital floor fractures resulting from MVCs (Fig. 8). Mandible fractures were most frequently associated with MVCs (36.6%) and assault-related injuries (21.3%) (Fig. 8). Additionally, firearm-related injuries were most strongly associated with mandible and maxilla/zygoma fractures (Supplemental Table 1).

**Fig. 9:**
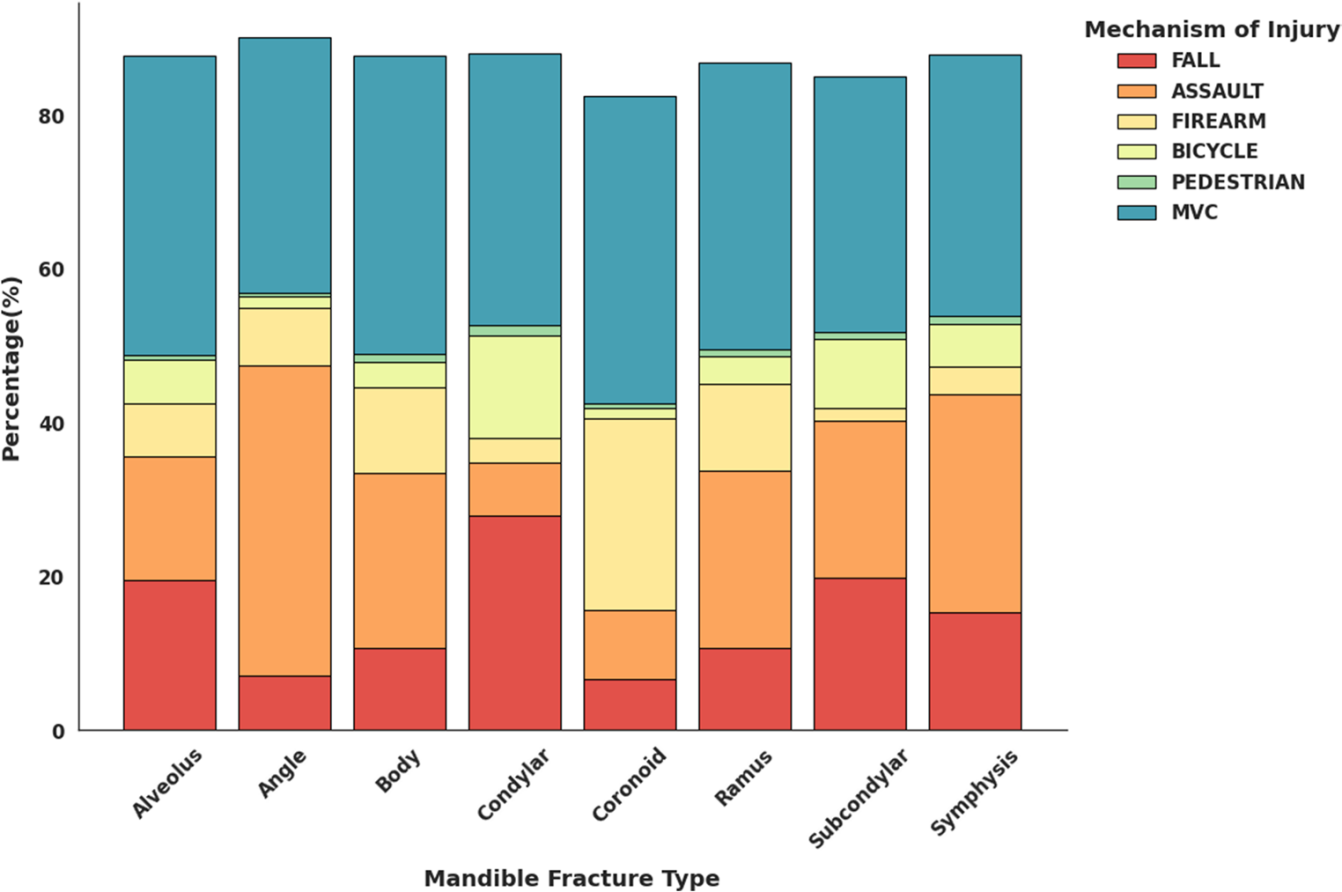
Distribution of mechanisms of injury stratified by mandibular fracture type

Mandibular fracture mechanisms varied significantly by anatomical region (χ² = 133.62, df = 40, *p* < 0.001). MVCs predominated across most subtypes, particularly in coronoid, alveolar, body, and ramus fractures, while falls were more commonly associated with condylar fractures. Assault-related injuries were most frequent in angle fractures, while firearm-related injuries peaked in coronoid fractures. Bicycle-related injuries were primarily linked to condylar fractures (Fig. 9).

**Fig. 10:**
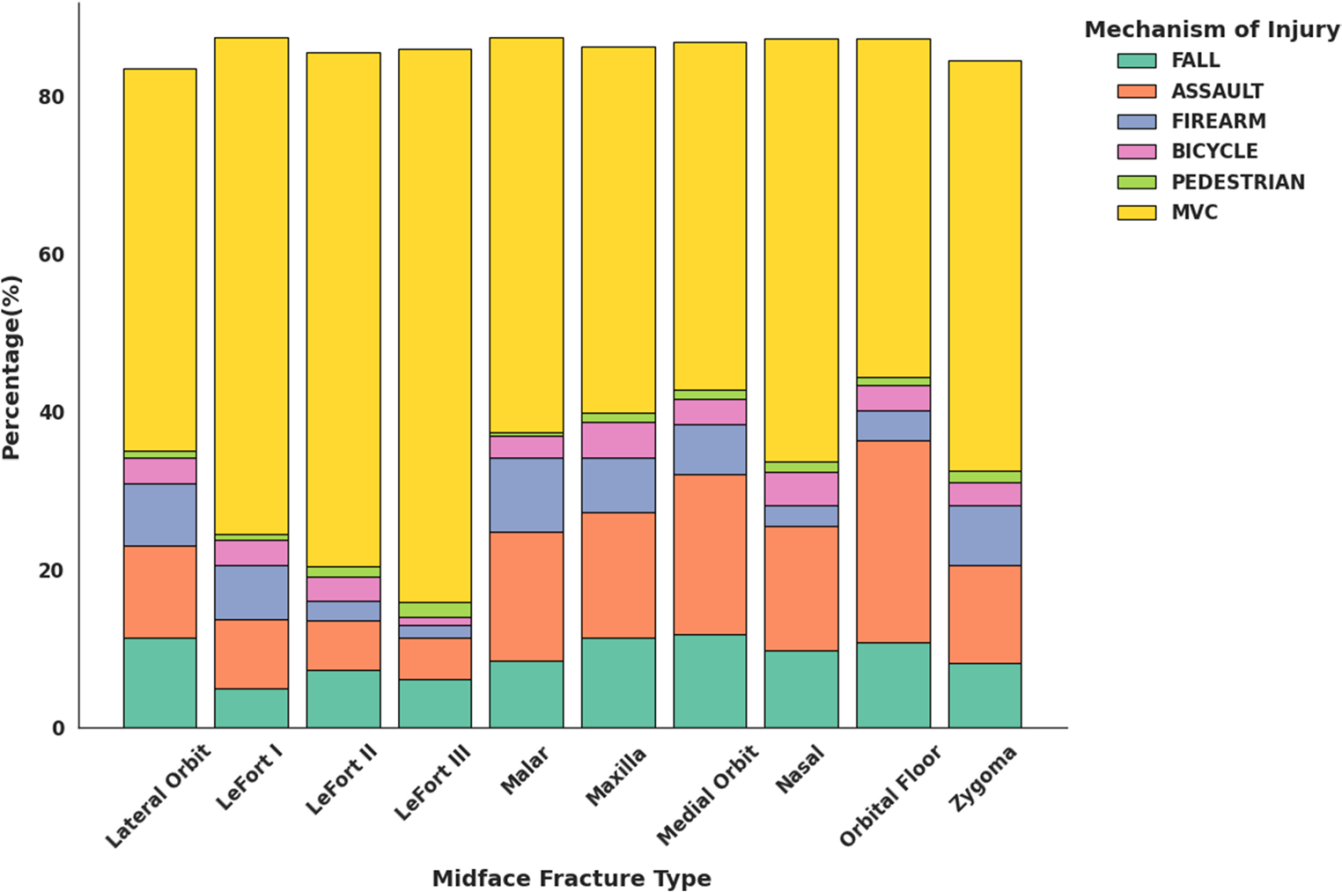
Distribution of mechanisms of injury stratified by midface fracture type

Midface fracture mechanisms also followed distinct patterns. MVCs were the leading cause among all subtypes, especially in LeFort fractures, while orbital floor fractures showed a lower MVC association (43.0%). Falls were primarily linked to maxillary and orbital fractures, while assault-related injuries disproportionately affected the orbital floor, medial orbit, and maxilla. Firearm-related injuries were most common in malar, lateral orbit, and zygoma fractures (Fig. 10).

## Discussion

This study represents the most extensive national evaluation to date of pediatric craniomaxillofacial trauma. Leveraging a comprehensive national dataset and contemporary medical coding systems, we identified rare fracture subtypes (e.g., LeFort fractures) and conducted a detailed analysis of injury mechanisms across developmental stages. Using the same age-stratification schema as Imahara et al., the first study of pediatric facial trauma using the NTDB, our findings provide a contemporary update and a national benchmark for evolving injury patterns and risk factors [9].

Our analysis confirms that high-energy trauma, particularly motor vehicle collisions, remains the primary cause of pediatric CMF fractures [9, 13, 20, 21]. Patients with CMF fractures were more likely to be older, male, and Caucasian compared to those without such injuries, consistent with previous national studies [9]. The risk of sustaining a CMF fracture was more than twice as high following MVCs compared to other injury mechanisms. Appropriate restraint use was associated with an approximately 43% reduction in fracture risk, a substantially greater effect than the 16% reduction reported by Imahara et al. [9]. Restraint compliance has also improved over time (60.3% in our study versus 31.9% previously) [9]. These gains likely reflect advances in restraint technology, vehicle safety, and public health messaging [22, 23].

Despite these improvements, the prevalence of MVC-related facial fractures has decreased only modestly since 2008 (24.2% in our study versus 29.9% previously) [9, 13]. Nearly 40% of children involved in MVCs in our cohort were unrestrained, and more than half of MVC-related fatalities occurred among these unrestrained children. This persistent gap may be attributed to several evolving risk factors. Improper restraint use—including incorrect installation or inappropriate selection of restraint devices—further contributes to injury risk, even among children who are technically restrained [22, 24–26]. Increasing driver distraction due to mobile device use has emerged as a significant contributor to pediatric and adolescent MVC injuries, potentially offsetting gains achieved through improved restraint technology and compliance [23, 27–34]. These findings highlight ongoing opportunities for targeted injury prevention and underscore the need for renewed public health strategies. Effective strategies should address not only restraint use, but also driver distraction and evolving risk behaviors, such as improper seat positioning and impaired driving among caregivers [31–34].

Age-related differences in injury mechanisms offer key insights into the changing risk profile of pediatric CMF trauma. In younger children, falls were the most common cause of injury, likely reflecting early motor development and limited exposure to high-energy hazards due to parental supervision [8, 35]. Older children and adolescents had a higher incidence of MVCs, likely owing to increased independence and greater vehicular exposure [4, 36]. Bicycle-related injuries peaked during mid-childhood and early adolescence, paralleling increased independent outdoor activities [36, 37]. Assault-related injuries became more prevalent with age, reflecting broader societal trends in youth violence [38, 39]. A rise in firearm-related injuries was observed among adolescents, consistent with previous reports linking teenage craniofacial trauma to gun violence [39].

Our age-stratified, mechanism-specific analysis underscores the importance of prevention strategies tailored to each developmental stage. For young children, home safety interventions targeting fall prevention have centered on the use of safety gates, furniture corner covers, and limiting baby walker use [40–42]. The most comprehensive systematic review to date by Young et al. found that while these interventions increase safety device use and reduce certain hazards, evidence for a direct reduction in falls or fall-related injuries remains limited and inconsistent [40]. McClure et al. conducted a systematic review of community-based programs and identified few high-quality studies and little conclusive evidence for a reduction in fall-related injury rates among children [41]. Promising results have emerged from recent large-scale, multicomponent interventions. The national Home Safety Equipment Scheme in England, which provided safety equipment and advice to families in deprived areas, was associated with a measurable reduction in hospital admissions for unintentional injuries among children under five [42]. Although not exclusively focused on falls, this reduction suggests that broad, comprehensive programs may contribute to fall prevention. Future efforts should build on such models and rigorously evaluate fall-specific outcomes.

Vehicle safety interventions—including booster seat legislation, seatbelt enforcement, and distracted driving prevention—have been shown to increase restraint use and reduce injuries among children and adolescents. Booster seats lower injury risk by 20–30% in children aged 4–12 years, and seatbelt laws and graduated driver licensing are associated with fewer adolescent fatalities and crash rates [42–44]. Community-based violence prevention and firearm safety interventions, especially multipronged programs such as hospital-based violence intervention and the Communities That Care trial, have reduced youth firearm injuries, risky behaviors, and handgun carrying by up to 24–27% [45–48]. Firearm safety counseling and provision of locking devices improve safe storage practices among families. Despite these advances, variable program effectiveness and ongoing disparities underscore the need for comprehensive, culturally tailored approaches that integrate policy, education, and community engagement to further reduce injury rates among youth.

Age-dependent trends in CMF fracture distribution are consistent with previous research, demonstrating a shift from cranial-dominant fracture patterns in younger children to increased facial fractures in adolescents [9, 19]. This transition likely reflects skeletal maturation, such as greater facial bone projection and paranasal sinus pneumatization [36, 49]. Cranial vault fractures predominated across all age groups except adolescents, in whom cranial base fractures were most common. This pattern likely reflects both developmental changes and increased exposure to high-energy MVCs [4, 13, 20]. The age-related increase in midface, mandibular, and dental fractures likely reflects both greater exposure to violent and high-energy mechanisms, as well as increased facial prominence with age [10, 50].

The distribution of mandibular fractures in our cohort is consistent with prior studies, with the body, condyle, and symphysis as the most commonly affected sites [51–54]. Approximately one in four mandibular fracture cases involved multiple fractures, most commonly in the condyle and symphysis. This pattern likely reflects indirect force transmission through the chin, the high cartilage content of the condyle, and the protective effect of developing dentition, which redirects stress away from the mandibular body [51–55]. The angle and symphysis were also frequently fractured together, likely due to biomechanical stress propagation and flexural loading that distribute forces between these two sites during trauma [51–53, 55, 56].

MVCs were the primary cause of coronoid, alveolar, and body fractures. High-impact forces in these collisions disproportionately affect structurally rigid regions of the mandible [50, 54]. The high prevalence of coronoid fractures in MVC-related trauma is attributed to indirect blunt force transmission during impact; the coronoid process, though protected by the zygoma, rarely fractures in isolation [50, 54]. The high frequency of alveolar fractures is likely due to direct impact on the dentoalveolar structures, which are particularly vulnerable in frontal collisions [43]. Body fractures in MVCs typically result from high-energy, direct impact forces (e.g., airbag deployment or dashboard impact), leading to localized stress concentration [56, 57]. Falls and bicycle-related trauma were disproportionately linked to condylar fractures, consistent with biomechanical models predicting that chin impacts preferentially transmit forces to the condylar and subcondylar regions [54, 55]. Assault-related injuries were the primary cause of angle fractures and were also highly prevalent in symphyseal and body fractures, reflecting the characteristic impact patterns of interpersonal violence [54–58]. Firearm-related injuries were most frequently associated with coronoid, ramus, and body fractures, likely due to the high-velocity impact of ballistic trauma, extensive energy transfer, cavitation effects, and bullet yawing [54–59].

The predominance of orbital fractures among midfacial fractures underscores the vulnerability of the pediatric orbital region. The orbital floor was the most frequently injured subsite, while the lateral wall was the least affected. This likely reflects the orbital floor’s thin structure, proximity to the maxillary sinus, and susceptibility to indirect force transmission in “blowout” fractures [51, 60]. In contrast, the lateral orbital wall is reinforced by the dense zygoma and sphenoid bones, providing structural support and resistance to injury [51]. Distinct patterns in midface fracture mechanisms were observed, but the overall association did not reach statistical significance, suggesting that factors such as anatomical variation or differences in impact dynamics may influence these patterns. MVCs were the primary mechanism for midface fractures, especially LeFort fractures, emphasizing the role of rapid deceleration and blunt impact in producing complex, multifragmentary facial injuries [60]. LeFort fractures were relatively rare, comprising only 3% of all CMF fractures. The pediatric facial skeleton is more resistant to high-energy trauma than the adult skeleton, largely due to the increased cranial-to-facial ratio, which helps dissipate impact forces away from the midface [60, 61]. A key strength of this study was the ability to identify LeFort fractures using modern clinical coding (ICD-10), a refinement not possible in studies based on ICD-9 codes [9]. However, individual codes for frontal sinus and naso-orbito-ethmoid fractures remain lacking in ICD-10, which may confound analysis of these fracture patterns.

The frequent occurrence of additional CMF fractures in approximately half of dentoalveolar fracture cases aligns with previous literature, suggesting that dentoalveolar trauma often results from high-impact forces capable of causing concurrent craniofacial injuries [62]. This underscores the importance of thorough evaluation in patients with dentoalveolar fractures to identify potential maxillofacial trauma. The bimodal distribution of isolated dentoalveolar fractures, with a primary peak between ages 10–11 and a secondary peak in children aged 2–3 years, suggests that different etiological factors are operative at different developmental stages [62]. The early peak likely reflects falls related to early mobility and lack of coordination in toddlers, whereas the later peak may result from sports-related trauma and increased physical activity in older children [62].

This study has several limitations. The analysis relies on the NTDB, a voluntary convenience sample rather than a comprehensive epidemiologic registry, which limits the generalizability of the findings to the broader pediatric trauma population. The NTDB also predominantly captures high-acuity trauma managed in resource-rich settings. The database provides only broad descriptive variables and lacks detailed patient-specific data—such as precise anatomical measurements, radiologic imaging, and narrative injury descriptions—necessary for a more nuanced assessment of injury severity. Reliance on ICD-10 coding, which is designed primarily for administrative and billing purposes, may result in misclassification of injuries and does not capture more refined fracture characteristics. Regional variations in safety regulation enforcement, seasonal conditions, and trauma referral patterns may significantly influence the distribution of injuries across regions and studies, further limiting direct comparability in registry-based analyses [22, 23, 27, 28]. Direct comparison with other epidemiological studies also remains challenging due to substantial heterogeneity in the anatomical sites included and pediatric age cut-offs. Other limitations include potential missing data and the retrospective study design, which precludes causal inferences. Despite these constraints, the study provides a valuable nationwide snapshot of pediatric CMF trauma and reinforces age-related variations in craniofacial trauma reported in previous studies [9, 13]. Future research should explore the use of machine learning to predict clinical decisions such as the likelihood of multiple CMF fractures, the need for open fixation, and other outcomes, enhancing personalized care. Studies incorporating detailed anatomical measurements, imaging data, and comprehensive narrative descriptions are also warranted to refine classification systems and validate these findings.

There is a clear need for age-specific, mechanism-oriented treatment algorithms and a multidisciplinary approach to care, particularly given the high prevalence of pediatric patients with multiple craniofacial injuries. The findings from this study should inform clinical practice, guide public health interventions, and provide a foundation for policy recommendations to improve the prevention and management of pediatric CMF trauma.

## Supplementary Information

Below is the link to the electronic supplementary material.

Supplementary Material 1

## Data Availability

The data that support the findings of this study are available from TQIP, but restrictions apply to the availability of these data, which were used under license for the current study, and so are not publicly available. Data are however available from the corresponding authors upon reasonable request and with permission of TQIP. The ACS TQIP database can be requested through the ACS TQIP (https://www.facs.org/quality-programs/trauma/quality/national-trauma-data-bank/datasets/). The dataset is free for institutions participating in the TQIP program and is available on reasonable request to the TQIP steering committee for a service fee for non-participating institutions through the same link.
