# Supplementary material for: Mechanism patterns and age variations in pediatric cranio-maxillofacial trauma: a 5-year analysis of the national trauma data bank
Source: Oral Maxillofac Surg. 2025 Sep 16;29(1):153. doi: 10.1007/s10006-025-01450-3 (PMC12441080; doi:10.1007/s10006-025-01450-3)
Supplement: Supplementary file 1 — Supplementary Material 1 [file 10006_2025_1450_MOESM1_ESM.docx]

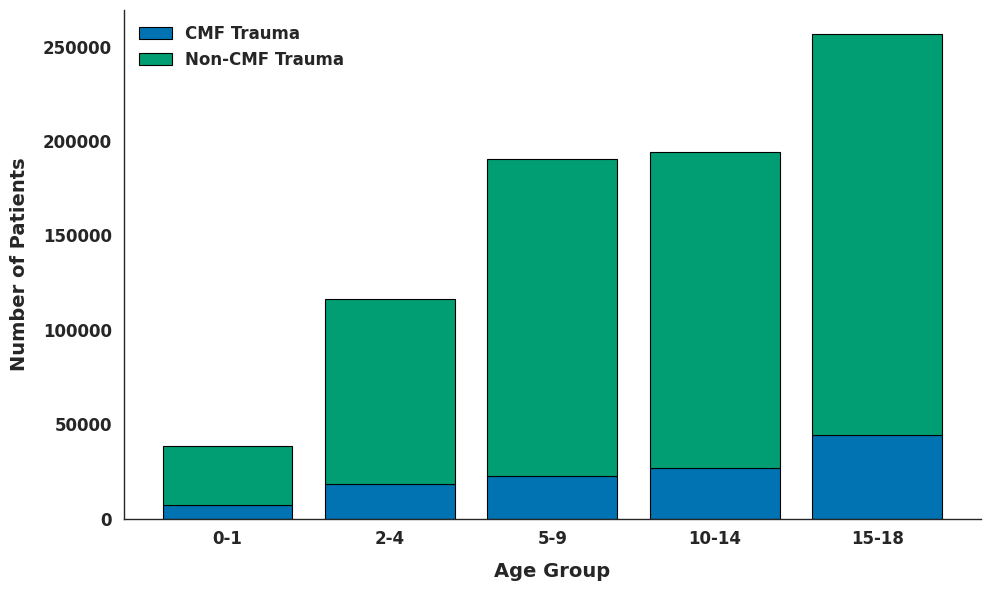


**Supplemental Figure 1.** Distribution of pediatric trauma cases by age group, separated into CMF and non-CMF trauma.

**Supplemental Table 1.** Distribution of CMF Fractures by Injury Mechanism

| **Fracture Type** | **Fall** | **Assault** | **Firearm** | **Bicycle** | **Pedestrian** | **MVC** |
| --- | --- | --- | --- | --- | --- | --- |
| **Cranial Base** | **12208 (30.5%)** | **3237 (8.08%)** | **2196 (5.48%)** | **1707 (4.26%)** | **765 (1.91%)** | **15017 (37.8%)** |
| **Cranial Vault** | **19,465 (39.1%)** | **4963.3668 (9.96%)** | **2880 (5.78%)** | **2088 (4.19%)** | **817 (1.64%)** | **14038 (28.2%)** |
| **Dental** | **2136 (18.3%)** | **1425 (12.2%)** | **541 (4.63%)** | **961 (8.23%)** | **214 (1.83%)** | **4998 (42.8%)** |
| **Nasal** | **1889 (9.72%)** | **3058 (15.7%)** | **537 (2.76%)** | **816 (4.20%)** | **247 (1.27%)** | **10410 (53.6%)** |
| **Mandible** | **3489 (14.83%)** | **5015 (21.3%)** | **1917 (8.15%)** | **1381 (5.87%)** | **242 (1.03%)** | **8603 (36.6%)** |
| **Alveolus** | **196 (19.5%)** | **161 (16.1%)** | **70 (6.96%)** | **57 (5.67%)** | **5 (0.540%)** | **392 (39.0%)** |
| **Angle** | **247 (7.19%)** | **1386 (40.2%)** | **257 (7.47%)** | **51 (1.49%)** | **17 (0.500%)** | **1148 (33.3%)** |
| **Body** | **609 (10.7%)** | **1305 (22.9%)** | **626 (11.0%)** | **192 (3.37%)** | **59 (1.04%)** | **2214 (38.8%)** |
| **Condylar** | **1312 (27.9%)** | **328 (6.99%)** | **144 (3.07%)** | **632 (13.4%)** | **60 (1.29%)** | **1663 (35.4%)** |
| **Coronoid** | **21 (6.71%)** | **28 (8.95%)** | **79 (24.9%)** | **4 (1.28%)** | **2 (0.640%)** | **128 (39.9%)** |
| **Ramus** | **260 (10.7%)** | **560 (23.1%)** | **274 (11.3%)** | **85 (3.51%)** | **23 (0.980%)** | **905 (37.3%)** |
| **Subcondylar** | **255 (19.9%)** | **261 (20.4%)** | **20 (1.62%)** | **115 (9.01%)** | **11 (0.900%)** | **427 (33.2%)** |
| **Symphysis** | **573 (15.40%)** | **1052 (28.3%)** | **136 (3.67%)** | **206 (5.54%)** | **40 (1.08%)** | **1265 (34.0%)** |
| **Maxilla** | **2189 (11.36%)** | **3068 (15.92%)** | **1320 (6.85%)** | **869 (4.51%)** | **223 (1.16%)** | **8949 (46.44%)** |
| **Malar** | **19 (8.41%)** | **36 (16.4%)** | **21 (9.35%)** | **6 (2.80%)** | **1 (0.470%)** | **113 (50.0%)** |
| **Zygoma** | **512 (8.19%)** | **778 (12.4%)** | **474 (7.58%)** | **177 (2.84%)** | **93 (1.50%)** | **3250 (51.9%)** |
| **Lateral Orbit** | **294 (11.3%)** | **305 (11.8%)** | **202 (7.81%)** | **83 (3.20%)** | **23 (0.920%)** | **1254 (48.3%)** |
| **Medial Orbit** | **529 (11.8%)** | **905 (20.2%)** | **280 (6.26%)** | **148 (3.31%)** | **48 (1.09%)** | **1972 (44.1%)** |
| **Orbital Floor** | **1399 (10.8%)** | **3317 (25.6%)** | **487 (3.76%)** | **409 (3.16%)** | **131 (1.01%)** | **5569 (43.0%)** |
| **Malar** | **19 (8.41%)** | **36 (16.4%)** | **21 (9.35%)** | **6 (2.80%)** | **1 (0.470%)** | **113 (50.0%)** |
| **LeFort I** | **35 (5.03%)** | **60 (8.62%)** | **48 (6.90%)** | **23 (3.30%)** | **5 (0.720%)** | **437 (62.8%)** |
| **LeFort II** | **60 (7.32%)** | **51 (6.22%)** | **21 (2.56%)** | **25 (3.05%)** | **10 (1.22%)** | **537 (65.1%)** |
| **LeFort III** | **51 (6.15%)** | **43 (5.19%)** | **14 (1.69%)** | **8 (0.970%)** | **16 (1.93%)** | **582 (69.9%)** |
